# Characterization of *Ziziphus lotus'* Activated Carbon and Evaluation of Its Adsorption Potential

**DOI:** 10.1155/2022/8502211

**Published:** 2022-04-25

**Authors:** Ibrahim Touzani, Kawtar Fikri-Benbrahim, Hammou Ahlafi, Bouchaib Ihssane, Otmane Boudouch

**Affiliations:** ^1^Laboratory of Microbial Biotechnology and Bioactive Molecules, Sciences and Technologies Faculty, Sidi Mohamed Ben Abdellah University, P.O. Box 2202, Fez, Morocco; ^2^High Institute of Nursing Professions and Health Techniques Annex Taza, Fez, Morocco; ^3^Laboratory of Chemistry and Biology Applied to the Environment, Faculty of Sciences, Moulay Ismail University, P. O. Box. 11201, Zitoune, Meknes 50060, Morocco; ^4^Laboratory of Applied Organic Chemistry Sciences and Technologies Faculty, Sidi Mohamed Ben Abdellah University, P.O. Box 2202, Fez, Morocco; ^5^Environmental & Agro-Industrial Processes Team, Sciences and Technologies Faculty, Sultan Moulay Slimane University, P. O. Box 523, Beni-Mellal, Morocco

## Abstract

This study aims to prepare activated carbon from an interesting biomaterial, corresponding to the cores of *Ziziphus lotus*, for the first time to the best of our knowledge, according to a manufacturing process based on its chemical and thermal activation. These cores were chemically activated by sulfuric acid for 24 h and then carbonized at 500°C for 2 hours. The obtained activated carbon was characterized by scanning electron microscopy, X-ray diffraction, Fourier transform infrared spectroscopy, and Brunauer–Emmett–Teller (BET) analysis. The adsorption of methylene blue (MB) on the activated carbon was evaluated, by Langmuir and Freundlich models examination, in order to explain the adsorption efficiency in a systematic and scientific way. Moreover, pseudo-first-order and pseudo-second-order kinetic models were used to identify the mechanisms of this adsorption process. The characterization results showed an important porosity (pore sizes ranging from 10 to 45 *µ*m), a surface structure having acid groups and carboxylic functions, and a specific surface of 749.6 m^2^/g. Results of the MB adsorption showed that this process is very fast as more than 80% of MB is adsorbed during the first 20 minutes. In addition, increasing the contact time and temperature improves the MB removal process efficiency. Moreover, this adsorption's kinetic modeling follows the pseudo-second-order model. Furthermore, data on the adsorption isotherm showed a maximum adsorption capacity of 14.493 mg/g and fit better with the Langmuir model. The thermodynamic parameters (∆G_0_, ∆S_0_, and ∆H_0_) indicate that the adsorption process is endothermic and spontaneous. Therefore, *Ziziphus lotus* can be used as a low-cost available material to prepare a high-quality activated carbon having a promising potential in the wastewater treatment.

## 1. Introduction

Water is the most fundamental and essential element of all natural resources. Its role is crucial for the economic and social development of a country; nevertheless, the management of water resources is a challenge for most countries in the world. Currently, these resources are facing problems of quantity and quality, related to global warming and irrational use. Thus, their quality is increasingly worsened by various pollutant discharges, such as urban and industrial wastewaters [1], leading to pollution of surface water, groundwater, and soil, which can directly affect ecosystems and the services they provide [2].

Wastewater can contain many substances, in solid or dissolved forms that are more or less biodegradable [3]. Among these organic micropollutants, dyes which are used in many industrial sectors such as textiles, food, and cosmetics, have particular characteristics such as their synthetic origin and a complex molecular structure that make them more stable and difficult to biodegrade [4]. The various dyes existing in industrial effluents can present adverse environmental effects, as they cause a change of the waters color, reducing sunlight penetration and photosynthetic activities [5]. As a result, ecological damage can spread downstream to agricultural or aquaculture areas, affecting aquatic flora and fauna [6]. In addition, they can also cause eye burns responsible for permanent injury to human and animal eyes, or waterborne diseases which could be considered carcinogenic and dangerous even at trace amounts [7]. Thereby, the treatment of industrial effluents containing different types of dyes is found to be of great interest to preserve our environment.

Various wastewater purification and treatment methods with technical, economic, and environmental advantages have been developed and used, such as dry desulfurization of MnOx hydrothermally loaded halloysite desulfurizer [8], vermifiltration recently identified as one of the best sustainable, natural, and ecofriendly technology for wastewater treatment [9], elimination of chlorinated VOCs by using HCl-tolerant HxPO_4_/RuOx-CeO_2_ catalysts [10]. Wetlands, considered to be inexpensive and having a wide variety of applications for domestic, agricultural, and industrial waters, can also be used for wastewater treatment [11], in addition to ozonation, precipitation, coagulation, flocculation, and adsorption. The latter method is the most widely used for the treatment of some micropollutants such as dyes [12, 13], trace metals [14], pesticides [15] and pharmaceuticals [16], thanks to its very significant technical, economic, and environmental benefits [17], and its high efficiency (90–99%) to remove soluble, insoluble, and biological contaminants.

Since the introduction of this process, activated carbon has been one of the most frequently used products due to its highly developed adsorption capacity. The main characteristics of activated carbon are its porous structure, linked to the accessible specific surface area into which molecules can penetrate, the pore size distribution, and their average geometric shape [18]. The adsorption properties of activated carbon depend on the functional groups, resulting mainly from the activation process, precursors, and thermal purification [19]. Therefore, the selection of activation agents remains a key issue for many researchers. Thus, several scientific papers report activation with potassium hydroxide [20, 21], phosphoric acid [22, 23], zinc chloride [24, 25], and new activation agents [26]. In addition, many authors have investigated the efficiency of inexpensive and available substances for the synthesis of activated carbon [27]. This has led to a growing interest in the production of activated carbon from renewable and low-cost precursors from agricultural waste. The most common activation sources on a commercial scale are wood, anthracite, bitumen charcoal, lignite, coconut shell, and almond shells. Today, much effort is devoted to the exploitation of industrial waste and agricultural residues as raw materials for coal production. Among its products are walnut shells [28], argan shells [29], snail shell [30], olive nibs [31], potato peel [32], sugar cane bagasse [33], date press cake [34], betel nuts [35], and prickly pear seeds [36].

This study concerns the preparation of an activated carbon from a biomaterial and its manufacturing process based on chemical and thermal activation. The biomaterial used in this study is the cores of *Ziziphus lotus* (Nbeg). To the best of the author's knowledge, no study has been conducted to investigate the feasibility of developing activated carbon from *Ziziphus* lotus which is very abundant and occupies various geographical areas of Morocco [37]. Indeed, this species is present in several biotopes of arid and semiarid regions. It grows on all soils: limestone, siliceous, clayey, and sandy [38]. Another part of this study concerns the use of this activated carbon as a new support for the depollution of urban and industrial wastewater loaded with organic pollutants such as dyes, in the aim to ecofriendly valorize agricultural by-products derived from the studied biomaterial by developing a more sustainable natural wastewater treatment technology. In this framework, an example of adsorption of methylene blue CI 52015 is evaluated, and its kinetic models are used to identify possible mechanisms of adsorption processes. Langmuir and Freundlich models are examined to explain the adsorption efficiency in a systematic and scientific manner.

## 2. Materials and Methods

### 2.1. Preparation of Activated Carbon

The preparation of our activated carbon follows several steps. The fruits of Nbeg: *Ziziphus lotus* wild (NZL) were harvested at full maturity in September 2018 in the region of Taza (Morocco). These fruits are characterized by a brown color and round shape; the kernels have an elongated shape, a light color, and a rough texture. The NZL pits were washed several times and then dried in shade away from dust, before being ground into fine particles. Chemical activation of NZL was carried out by sulfuric acid (H_2_SO_4_, 98%) for 24 h with mass contribution (1 : 1). Then, the resulting paste was washed with distilled water until the washing solution reached a neutral pH value, and then dried at 100°C for four hours. Then, the dried paste was carbonized at a temperature of 500°C for two hours. The resulting activated carbon was ground into small particles having sizes less than 100 *µ*m and named CNZL. The characterization of CNZL was performed using different analytical techniques: scanning electron microscopy (SEM) identified by Quanta 200 model SEM equipped with a tungsten filament electron gun; X-ray diffraction (XRD) performed by XPERT-PRO type XRD in a scan area ranging from 5 to 120 2*θ*. Fourier transform infrared spectroscopy (FTIR) was performed in the mid-infrared region using a Vertex 70 spectrometer. Specific surface area and pore structure were determined using an ASAP Micromeritics apparatus by adsorption of liquid nitrogen N_2_ at T = −196°C using the Brunauer–Emmett–Teller (BET) method and the Barrett–Joyner–Halenda (BJH) method.

### 2.2. Adsorption of CI 52015 Dye

#### 2.2.1. Adsorbate Preparation

**Figure 1:**
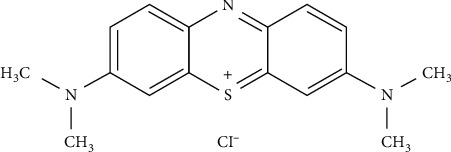
Molecular structure of methylene blue (CI 52015).

The pollutant considered in our study is the methylene blue (MB) dye: CI 52015 (Figure 1).

Solutions of different concentrations of 20, 30, and 40 mg/L were obtained by diluting a stock solution of MB (at 5 g/L) with distilled water.

#### 2.2.2. Effect of Contact Time

The contact time is one of the most important factors during chemical reactions. Hence, its effect on the removal rate of MB was studied for a time interval of 10–120 min for each initial concentration (20, 30, and 40 mg/L), in the presence of CNZL activated carbon at room temperature. So, in closed reactors, 100 ml of each methylene blue concentration was put in contact with 0.1 g of CNZL activated carbon under stirring at 300 rpm. Every 10 min, the mixture was centrifuged at 5000 rpm for 5 min, and then the concentrations of MB in the supernatants were measured at 665 nm using an UV-Visible spectrometer.

The abatement rate *R* (%) was calculated from the following equation:

(1) $$\begin{array}{c} R\left(\%\right)=\frac{C_{0}-C_{e}}{C_{0}}\times 100, \end{array}$$

where *C*_*0*_ (mg/L) is the initial concentration of MB and *C*_e_ (mg/L) is the MB concentration in solution.

#### 2.2.3. Effect of Temperature

To evaluate the effect of temperature on the adsorption capacity of MB on the CNZL adsorbent in a batch system, 0.1 g of activated carbon at a concentration of 20 mg/L was mixed with MB at 25 mg/L for 60 minutes, at different temperatures (25–50°C). Then, the residual concentrations (*Ce*) of each adsorbate were determined as explained above.

#### 2.2.4. Kinetics of Adsorption

Kinetics provides information about the adsorption mechanism and the mode of transfer of solutes from the liquid to the solid phase. Hence, simplified kinetic models are adopted to provide information about the adsorption mechanism. These models are generally well fitted by two classical kinetic models, a pseudo-first order kinetic model [39] and a pseudo-second-order kinetic model [40]. These two models were tested to investigate the adsorption kinetics of methylene blue on CNZL activated carbon.

*(1) Pseudo-First Order*. This model is defined by the following relationship [41]:

(2) $$\begin{array}{c} \log\;\;\left(q_{e}-q_{t}\right)=\log\;q_{e}-\frac{K_{1}\cdot t}{2.303}, \end{array}$$

where *q*_e_ and *q*_*t*_ are the quantities of adsorbed ion (mg·g^−1^) at equilibrium and time *t*, respectively, and *k*_*1*_ is the equilibrium velocity constant of pseudo first order (min^−1^).

*(2) Pseudo-Second Order*. This model is defined in the following form [42]:

(3) $$\begin{array}{c} \frac{t}{q_{t}}=\frac{1}{K_{2}q_{e}^{2}}+\frac{t}{q_{e}}, \end{array}$$

where *k*_*2*_ is the pseudo-second-order adsorption rate constant (g/mg·min).

#### 2.2.5. Adsorption Isotherms

An adsorption process can be described using an adsorption isotherm. Langmuir and Freundlich isotherms are used as models to study the adsorption of MB on CNZL activated carbon. An isotherm is a curve that represents the relationship between the amount of solute adsorbed per unit mass of adsorbent *q*_*e*_ and the concentration of solute in solution *C*_*e*_.

The amount of solute adsorbed is calculated using the following equation [43]:

(4) $$\begin{array}{c} q_{e}=\frac{\left(C_{0}-C_{e}\right)\cdot V}{m}, \end{array}$$

where *q*_*e*_ is the equilibrium amount of solute adsorbed per unit weight of adsorbent (mg/g); C_0_ is the initial solute concentration (mg/L); Ce is the equilibrium solute concentration (mg/L); m is the mass of adsorbent (g); V is the volume of solution (L).

Langmuir model [44] is described mathematically by the following equation:

(5) $$\begin{array}{c} q_{e}=\frac{q_{\max}K_{L}C_{e}}{1+K_{L}C_{e}}. \end{array}$$

This equation is often written in linear form [45]:

(6) $$\begin{array}{c} \frac{C_{e}}{q_{e}}=\frac{1}{K_{L}q_{\max}}+\frac{1}{q_{\max}}C_{e}, \end{array}$$

where *q*_*e*_ and *q*_max_ are, respectively, the equilibrium adsorption capacity and the maximum adsorption capacity (mg/g), *K*_*L*_ the characteristic constant of Langmuir, and it is the equilibrium solution adsorbate concentration per unit mass of solid (mg/L). The adsorption characteristics of the Langmuir isotherm can be explained in terms of a nondimensional constant *R*_*L*_ [46].

The adsorption will be favorable if 0 < *R*_*L*_ < 1, unfavorable if *R*_*L*_ > 1; and if *R*_*L*_ = 0: the process will be irreversible [47].

Freundlich model [48] is described by the following empirical equation:

(7) $$\begin{array}{c} q_{e}=K_{f}*C_{e}^{1/n}. \end{array}$$

It can be expressed in logarithmic form according to [49]

(8) $$\begin{array}{c} \log\;q_{e}=\log\;k_{f}+\frac{1}{n}\log\;C_{e}, \end{array}$$

where *q*_*e*_ is the amount of adsorbate material adsorbed per unit mass of adsorbent at equilibrium (mg/g). This is the equilibrium concentration (mg/L), and *K*_*f*_ and *n* are, respectively, the adsorbent adsorption capacity and the adsorbent-adsorbate sorption intensity.

#### 2.2.6. Thermodynamic Study of Adsorption

Thermodynamic parameters such as thermodynamic equilibrium constant K_d_, Gibbs free energy variation *ΔG*_*0*_, enthalpy variation *ΔH*_*0*_, and entropy variation *ΔS*_*0*_ are among the most important parameters involved in the establishment of an adsorption system, thus to evaluate the feasibility and nature of the adsorption process [50]. The thermodynamic parameters were obtained from the following equations [51]:

(9) $$\begin{array}{c} K_{d}=\frac{q_{e}}{C_{e}}, \end{array}$$

(10) $$\begin{array}{c} \Delta G_{0}=\Delta H_{0}-T\Delta S_{0}, \end{array}$$

where *k* is the equilibrium constant (L/mg), *R* is the gas constant (8.314 J/mol.K), and *T* is the absolute temperature (K).

Plotting ln *K*_*d*_ as a function of 1/*T* yields a line with a slope equal to *−∆H*_*0*_*/R* and an intercept equal to *∆S*_*0*_*/R.* Subsequently, the thermodynamic parameters of adsorption are calculated: standard free enthalpy (*∆G*_*0*_), standard enthalpy (*∆H*_*0*_), and standard entropy (*∆S*_*0*_).

## 3. Results and Discussion

### 3.1. Characterization of Materials

#### 3.1.1. Scanning Electron Microscopy (SEM)

**Figure 2:**
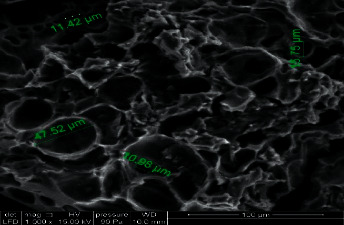
Image of the CNZL observed by Scanning Electron Microscopy (SEM).

The purpose of the SEM examination is to illustrate the morphology and external porosity of the activated carbon. A developed porosity allows increasing the specific surface and therefore the number of active sites on which the MB molecules can eventually bind.  Figure 2 shows a very porous morphology of CNZL activated carbon with pores of different sizes ranging from 10 to 45 *µ*m and different shapes.

#### 3.1.2. X-Ray Diffraction (XRD)

**Figure 3:**
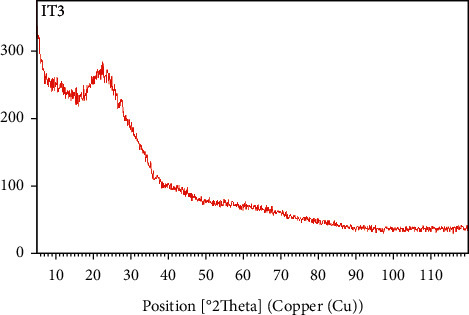
X-ray diffraction of the CNZL.

XRD is used to identify the crystalline or amorphous structure the CNZL.  Figure 3 of the X-ray diffraction (XRD) shows very broad diffraction peaks, with a broad peak at approximately 2*θ* = 25 indicating the presence of carbon. Thus, the absence of a sharp peak reveals a predominantly amorphous structure [52]. Furthermore, there are noise signals consistent with the ash powder of the activated carbon.

#### 3.1.3. Fourier Transform Infrared Spectroscopy (FTIR)

**Figure 4:**
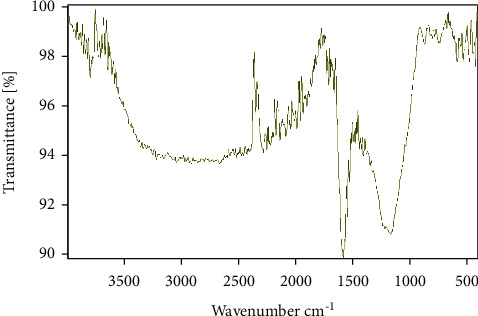
FTIR spectrum of the CNZL.

Infrared spectroscopy is an analytical technique that is related to the vibrational properties of interatomic bonds; it can identify functional groups present in molecules. The infrared spectrum of CNZL illustrated in Figure 4 shows many functions on the surface of activated carbon, similar to those found in lingo-cellulosic materials [53].

The adsorption bands are variable and narrow. The bands observed between 3700 and 3600 cm^−1^ correspond to the elongation vibrations of the free O-H bond of the alcoholic and phenolic groups, and the peak at 3640 cm^−1^ is attributed to the existence of free hydroxides [54]. The bands between 3305 and 3200 cm^−1^ correspond to the hydrogen elongation vibrations of the hydroxyl groups and water [55]. The bands observed between 2850 and 2930 cm^−1^ correspond to the symmetric and asymmetric valence vibration of the CH_2_ bonds. The bands in the range 2200–2000 cm^−1^ correspond to the C≡C bond valence vibration of the alkyl function [56]. The peak at 1680 cm^−1^ corresponds to the elongation vibration of C═C bonds in aromatic rings [57]. A strong prominent band in the activated carbon appeared at 1575 cm^−1^ and corresponds to the presence of the carboxylic C=O groups on the surface [58]. The bands observed in the 1400–1300 cm^−1^ region correspond to CH_2_ deformation and/or O-H deformation vibrations supported by the existence of phenols [59]. A broad band exists between 1200 and 1100 cm^−1^ and corresponds to the valence vibration of C-O in the acid, alcohol, and phenol groups, the peak at 1195 cm^−1^ is due to the S═O sulfur compounds present in the activated carbon. The band at 845 cm^−1^ as well as the bands that appear between 770 and 400 cm^−1^ represent vibrational distortion of C-H groups in aromatic rings [60].

#### 3.1.4. BET Analysis

**Figure 5:**
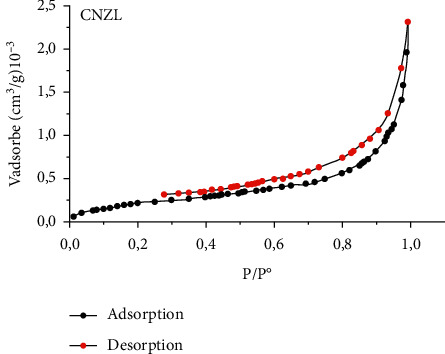
Adsorption/desorption isotherm of CNZL.

**Figure 6:**
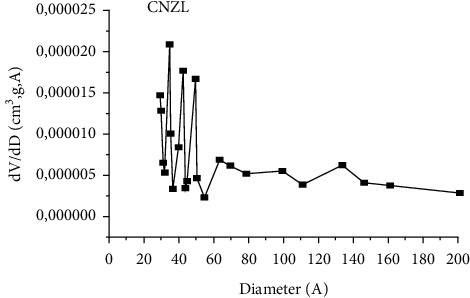
Pore diameter distribution of the CNZL.

The structure of an adsorbent is well defined by its specific surface area which represents the total surface area per unit mass of the product accessible to atoms and molecules, as well as the pore volume, the pore shape, and the pore size distribution [61].  Figure 5 shows a nitrogen adsorption/desorption isotherm obtained in the CNZL adsorbent. The adsorption curve obtained shows the characteristics of the type IV isotherm, where the surface of the adsorbent is completely covered by a monomolecular layer of N_2_ (B-point method), then beyond, several molecular layers (physisorption) are formed (according to the IUPAC classification) corresponding to mesoporous solids with capillary condensation. This is confirmed by the calculation of the pore size distribution, determined by the Barrett–Joyner–Halenda (BJH) method (Figure 6). This pore distribution shows average diameters between 20 and 60 Å. In addition, the specific surface area determined using the BET method is 749.6 m^2^/g for CNZL with a pore volume of 21.84 cm³/g.

### 3.2. Effect of Contact Time

**Figure 7:**
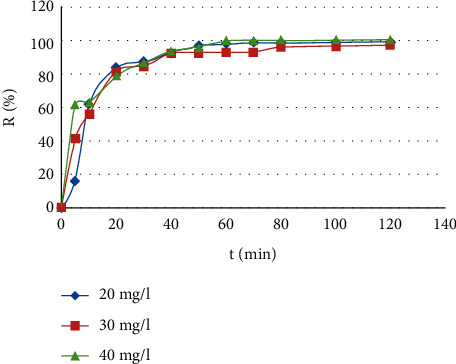
Removal rate of IC 52015 by CNZL. C = 20, 30, 40 mg/l *m* = 0.1 g.

Contact time is one of the most important factors in chemical reactions. In the adsorption process, the increasing of the adsorbate concentration in the solution leads to the adsorbates*'* introduction into the internal pores of the adsorbent due to the concentration gradient [19].  Figure 7 shows the removal rate of different concentrations of methylene blue (20, 30, and 40 mg/L) over time, by CNZL activated carbon adsorption. Results show that the adsorption process is very fast and the removal efficiency of MB is improved by increasing the contact time and depends on initial concentration. This evolution of the degradation efficiency over time was expected as it was revealed in other studies [62, 63]. Indeed, more than 80% of the dye used amount is adsorbed during the first 20 min, which could be due to the external mass transfer that is fast. Therefore, the increase in contact time greatly increases the removal efficiency and enables to reach equilibrium at about 80 min for the tested concentrations. This means that there is an internal mass transfer of the adsorbent, which generally corresponds to a diffusion phenomenon in the internal porosity.

### 3.3. Effect of Temperature

**Figure 8:**
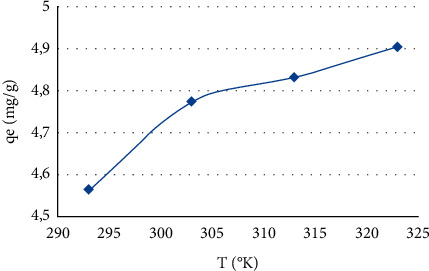
Effect of temperature on the adsorption capacity of MB. *T* = 1 h, m (CNZL) = 0.1 g, and [MB] = 25 mg/l.

The effect of solution temperature on MB dye removal was performed to determine the effect of temperature change on the equilibrium capacity of the adsorbent. The study was performed in the range of 293–323 K at an initial dye concentration of 25 mg/l and 0.1 g of CNZL. The results of the temperature effect suggest that the adsorption capacity of MB increases with increasing temperature (Figure 8), indicating that the process is endothermic according to Miraboutalebi et al. [64].

The increase in temperature allows the MB molecules to escape from the adsorbent and reenter the liquid phase because the mobility of the dye molecules increased with temperature [65]. This phenomenon acquires enough energy to interact with the active sites on the surface [13], which is also attributed to the creation of new active sites at higher temperature or the increased penetration of MB molecules into the porous structure of the CNZL carbon.

### 3.4. Adsorption Kinetics

**Figure 9:**
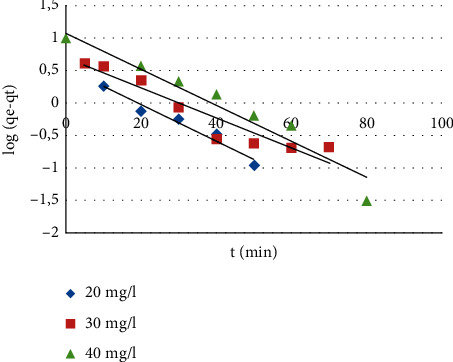
Pseudo-first-order kinetic model.

**Figure 10:**
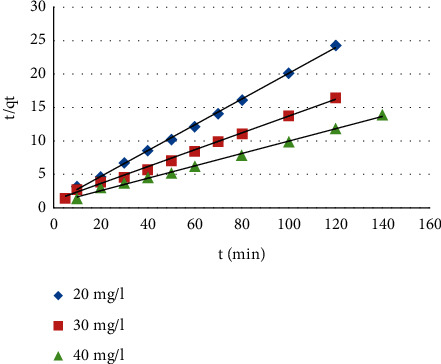
Pseudo-second-order kinetic model.

The correlation coefficients as well as the kinetic constants corresponding to the two models used are determined by plotting the log (*q*_*e*_−*q*_*t*_) versus time (*t*) for the pseudo-first-order model (Figure 9) and the (*t*/*q*_*t*_) versus time (*t*) for the pseudo-second-order model (Figure 10). The parameters for the two models of the MB's adsorption at different initial concentrations on CNZL activated carbon are grouped in Table 1. These results show that the *R*^2^ values are 0.954, 0.904, and 0.928 for pseudo-first order; and 0.999, 0.996, and 0.997 for the pseudo-second order, respectively, for MB concentrations of 20, 30, and 40 mg/L; which reveal that the correlation coefficients *R*^2^ of the pseudo-first-order model are lower than those obtained for the pseudo-second-order model, which are very close to unity for the adsorption of MB on CNZL. Furthermore, values of the experimental adsorption capacity and those of the calculated adsorption capacity of the pseudo-second-order model are very close (Table 1), which reflects a good fit of this model to explain the adsorption process of MB on CNZL. Similarly, it can be noticed that increasing the initial MB concentration leads to a decrease in the value of the rate constant *K*_*2*_, which can be attributed to strong competition for sorption sites at high concentration leading to higher sorption rates [21]. These results suggest that the kinetic data of MB adsorption on CNZL are better represented by the pseudo-second-order model.

### 3.5. Adsorption Isotherms

**Figure 11:**
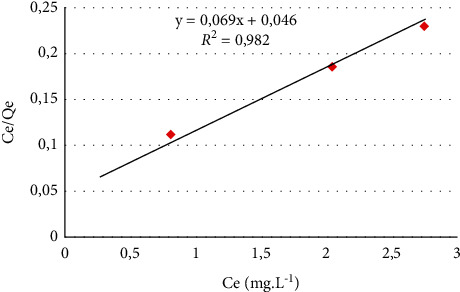
The Langmuir model representation.

**Figure 12:**
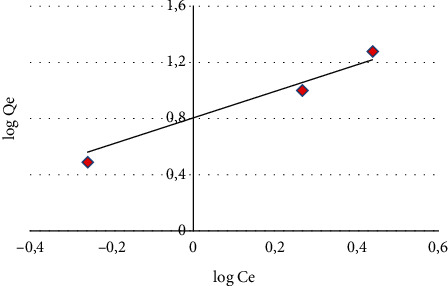
The Freundlich model representation.

Adsorption isotherms are intended to describe how adsorbates interact with adsorbents and are essential for optimizing the use of adsorbents [66]. These isotherms allow us to determine the adsorption capacity and to highlight whether or not purification is feasible. To define the model to which the adsorption of MB is subjected, the experimental data have been applied to equations of the two mathematical models of Langmuir and Freundlich. The Langmuir model is represented by the plot of *C*_*e*_*/q*_*e*_ versus *C*_*e*_ (Figure 11) and the Freundlich model is represented by the plot of log *q*_*e*_ versus log C_e_ (Figure 12), that give a linear graph with a slope of 1/*n* and the intercept with the *x*-axis give log *K*_*F*_, from which *n* and *K*_*F*_ can be, respectively, calculated. The validity of the experimental results depends on the correlation coefficient value *R*^2^: the closer the coefficient is to unity, the more the results are consistent with the model considered.  Table 2 summarizes the results of both isotherms. The *R*^2^ correlation coefficient values for the Langmuir and Freundlich model are 0.982 and 0.899 respectively; moreover, the maximum adsorption capacity is 14.493 mg/g. These results show that the Langmuir model has an *R*^2^ value close to unity and higher than that of the Freundlich model, suggesting that the Langmuir model has a good fit to experimental data of MB adsorption on CNZL and that the adsorption occurs in monolayer and involves independent identical sites in limited number according to Yang et al. [67]. The adsorption characteristics of the Langmuir *R*_*L*_ isotherm also have values between 0 and 1, which confirm that the adsorption process of MB on CNZL is favorable.

### 3.6. Thermodynamic Study of Adsorption

**Figure 13:**
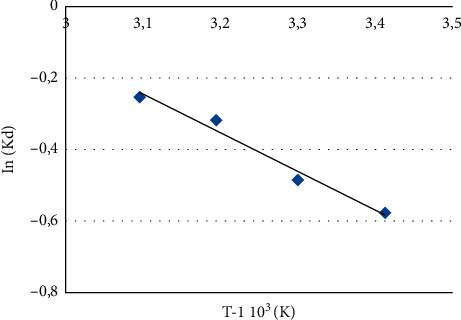
Evolution of Ln (*K*_*d*_) versus T^−1^.10^3^ for the adsorption of MB. *T* = 1 h, m (CNZL) = 0.1 g, and [MB] = 25 mg/l.

Thermodynamic parameters, such as thermodynamic equilibrium constant *K*_*d*_, Gibbs energy change *(ΔG*_*0*_), standard enthalpy change (*ΔH*_*0*_), and entropy change (*ΔS*_*0*_) are of great importance to study the feasibility and spontaneity of the adsorption process. The plot of ln *K*_*d*_ versus *T*^*−*1^ is shown in Figure 13, and the thermodynamic parameters were obtained from the slope and intercept of the plot of ln *K*_*d*_ versus 1/*T* and are presented in Table 3. This study reveals negative values of *ΔG*_*0*_ indicating that the adsorption of MB dye on CNZL is spontaneous and thermodynamically favorable. In general, the free energy change for physisorption is between −20 and 0 kJ/mol, but chemisorption is between −80 and −400 kJ/mol [68]. Therefore, the obtained values of *ΔG*_*0*_ are all less than 10 kJ/mol, suggesting that the adsorption of the MB dye on CNZL is physical in nature. In addition, the positive values of *∆H*_*0*_ show that the adsorption is endothermic and supported by the increase in dye adsorption capacity with increasing temperature. Moreover, the positive values of *∆S*_*0*_ show the affinity of the adsorbent for the MB and the increase in randomness at the solid-liquid interface.

## 4. Conclusion

This study was carried out in order to prepare an activated carbon from a biomaterial by using a manufacturing process based on chemical and thermal activations. The chemical activation was carried out by sulfuric acid (H_2_SO_4_, 98%) with a mass contribution (1 : 1), and the carbonization was conducted at a temperature of 500°C during 2 h. Characterization results by scanning electron microscopy and Fourier transform infrared spectroscopy reveals the presence of a porous structure having different functions on the surface of CNZL. Moreover, results show that the adsorption process is very fast and that the adsorption isotherm data are well fitted with the Langmuir monolayer model. Kinetic modeling of MB adsorption on CNZL activated carbon follows the pseudo-second-order model well. Hence, the peculiarity of this study, which aims to activate the fruits of a plant that is very well known locally, available but poorly valued have been well demonstrated. Furthermore, the obtained results show that the nuclei of *Ziziphus lotus* (Nbeg) represent an interesting source of raw material for the preparation of high-quality activated carbon and proved that this carbon: CNZL can be used as a new low-cost carrier for the remediation of urban and industrial wastewater loaded with organic pollutants such as dyes. Finally, this carbon should be promoted and considered as a cheaper alternative to commercial adsorbents. However, a pilot study is planned to better exploit this raw material which opens large industrial application perspectives and justify largely the economic impact of this study. This study also provided new perspectives on the valorization of agricultural by-products such as the cores of *Ziziphus lotus* for the preparation of activated carbon.

## Figures and Tables

**Table 1:** Adsorption kinetics parameters.

	*q*_*e*_ exp (mg·g^−1^)	Pseudo-first order			Pseudo-second order		
		*q*_*e*_ cal (mg·g^−1^)	*k*_*1*_ (min^−1^)	*R* ^2^	*q*_*e*_ cal (mg·g^−1^)	*K*_*2*_ (g·mg^−1^·min^−1^)	*R* ^2^
20 mg/L	4.935	3.411	0.064	0.954	5.23	0.037	0.999
30 mg/L	7.197	4.931	0.053	0.904	7.93	0.014	0.996
40 mg/L	9.996	12.05	0.062	0.928	10.86	0.009	0.997

**Table 2:** Parameters of the adsorption isotherm.

	Langmuir			Freundlich		
	*q*_max_ (mg/g)	*K*_*L*_ (L/mg)	*R* ^2^	*K*_*F*_ (mg.g^−1^) (L.mg^−1^)^1/n^	1/*n*	*R* ^2^
CNZL	14.493	1.50	0.982	6.45654229	0.926	0.899

**Table 3:** Thermodynamic parameters of MB adsorption on CNZL.

Dye	*R* ^2^	*ΔH*_*0*_ (kJ/mol)	*ΔS*_*0*_ (kJ/mol.K)	*ΔG*_*0*_ (kJ/mol)			
				298 K	303 K	313 K	323 K
MB	0.983	40.09	143.41	−41.97	−43.41	−44.84	−46.28

## Data Availability

All data generated or analyzed during the current study are available from the corresponding author on reasonable request.

## Supplementary Materials

Supplementary Materials We point out that it concerns an international patent including only a part of the results presented in our article and concerning the characterization of activated carbon only by electronic microscopy, diffraction of RX, and infrared red spectrum, with the reduction of methylene blue. The paper also includes the specific surface by the BET method and the effect of temperature, besides kinetic and thermodynamic studies of methylene blue adsorption and the Langmuir and Freundlich isotherms to determine the adsorption nature. The OMPIC file also concerns a national patent (presented in Morocco), including the same parameters presented for the WIPO one, and can be searched in the link of OMPIC (https://patent.ompic.ma/search) by giving the number of the patent (MA 49232).

## References

[1] Touzani I., Machkor M., Boudouch O., El Machrafi I., Flouchi R., Fikri-Benbrahim K. (2020). *Environmental Impact Assessment of Taza City’s Wastewater: Application through Principal Component Analysis*.

[2] Corcoran E. (2010). *Sick Water: The Central Role of Wastewater Management in Sustainable Development: a Rapid Response Assessment*.

[3] Alsheyab M., Kusch-Brandt S. (2018). Potential recovery assessment of the embodied resources in Qatar’s wastewater. *Sustainability*.

[4] Ghaedi M., Ghazanfarkhani M. D., Khodadoust S., Sohrabi N., Oftade M. (2014). Acceleration of methylene blue adsorption onto activated carbon prepared from dross licorice by ultrasonic: equilibrium, kinetic and thermodynamic studies. *Journal of Industrial and Engineering Chemistry*.

[5] Sikdar D., Goswami S., Das P. (2020). Activated carbonaceous materials from tea waste and its removal capacity of indigo carmine present in solution: synthesis, batch and optimization study. *Sustainable Environment Research*.

[6] Abbas M., Trari M. (2020). Removal of methylene blue in aqueous solution by economic adsorbent derived from apricot stone activated carbon. *Fibers and Polymers*.

[7] Mansour H. B., Boughzala O., Dridi D., Barillier D., Chekir-Ghedira L., Mosrati R. (2011). Les colorants textiles sources de contamination de l’eau: CRIBLAGE de la toxicité et des méthodes de traitement. *Revue des Sciences de l’Eau*.

[8] Lei Z., Hao S., Yusu W., Yang J. (2022). Study on Dry Desulfurization Performance of MnOx Hydrothermally Loaded Halloysite Desulfurizer. *Environmental Technology & Innovation*.

[9] Singh R., Samal K., Dash R. R., Bhunia P. (2019). Vermifiltration as a sustainable natural treatment technology for the treatment and reuse of wastewater: a review. *Journal of Environmental Management*.

[10] Dai Q., Shen K., Deng W. (2021). HCl-tolerant HxPO4/RuOx-CeO2 catalysts for extremely efficient catalytic elimination of chlorinated VOCs. *Environmental Science & Technology*.

[11] Wong A. (2017). Natural treatment technology for cleaning wastewater. *Water Conservation and Management*.

[12] Abbas M., Harrache Z., Trari M. (2019). Removal of gentian violet in aqueous solution by activated carbon equilibrium, kinetics, and thermodynamic study. *Adsorption Science & Technology*.

[13] Daoud M., Benturki O., Girods P., Donnot A., Fontana S. (2019). Adsorption ability of activated carbons from Phoenix dactylifera rachis and Ziziphus jujube stones for the removal of commercial dye and the treatment of dyestuff wastewater. *Microchemical Journal*.

[14] Betsholtz A., Karlsson S., Svahn O., Davidsson A., Cimbritz M., Falås P. (2021). Tracking 14C-labeled organic micropollutants to differentiate between adsorption and degradation in GAC and biofilm processes. *Environmental Science & Technology*.

[15] Gupta V. K., Gupta B., Rastogi A., Agarwal S., Nayak A. (2011). Pesticides removal from waste water by activated carbon prepared from waste rubber tire. *Water Research*.

[16] Yu F., Li Y., Han S., Ma J. (2016). Adsorptive removal of antibiotics from aqueous solution using carbon materials. *Chemosphere*.

[17] Bagheri A. R., Ghaedi M., Asfaram A. (2016). Modeling and optimization of simultaneous removal of ternary dyes onto copper sulfide nanoparticles loaded on activated carbon using second-derivative spectrophotometry. *Journal of the Taiwan Institute of Chemical Engineers*.

[18] Li X., Qiu J., Hu Y. (2020). Characterization and comparison of walnut shells-based activated carbons and their adsorptive properties. *Adsorption Science & Technology*.

[19] Yousefi M., Arami S. M., Takallo H. (2019). Modification of pumice with HCl and NaOH enhancing its fluoride adsorption capacity: kinetic and isotherm studies. *Human and Ecological Risk Assessment: An International Journal*.

[20] Wang Y. X., Ngo H. H., Guo W. S. (2015). Preparation of a specific bamboo based activated carbon and its application for ciprofloxacin removal. *Science of the Total Environment*.

[21] Abbas A. F., Ahmed M. J. (2016). Mesoporous activated carbon from date stones (Phoenix dactylifera L.) by one-step microwave assisted K_2_CO_3_ pyrolysis. *Journal of Water Process Engineering*.

[22] Kyzas G. Z., Deliyanni E. A., Matis K. A. (2016). Activated carbons produced by pyrolysis of waste potato peels: cobalt ions removal by adsorption. *Colloids and Surfaces A: Physicochemical and Engineering Aspects*.

[23] Han Q., Wang J., Goodman B. A., Xie J., Liu Z. (2020). High adsorption of methylene blue by activated carbon prepared from phosphoric acid treated eucalyptus residue. *Powder Technology*.

[24] Erdem M., Orhan R., Şahin M., Aydın E. (2016). Preparation and characterization of a novel activated carbon from vine shoots by ZnCl2 activation and investigation of its rifampicine removal capability. *Water, Air, & Soil Pollution*.

[25] Spagnoli A. A., Giannakoudakis D. A., Bashkova S. (2017). Adsorption of methylene blue on cashew nut shell based carbons activated with zinc chloride: the role of surface and structural parameters. *Journal of Molecular Liquids*.

[26] Heidarinejad Z., Dehghani M. H., Heidari M., Javedan G., Ali I., Sillanpää M. (2020). Methods for preparation and activation of activated carbon: a review. *Environmental Chemistry Letters*.

[27] Liu Q. X., Zhou Y. R., Wang M. (2019). Adsorption of methylene blue from aqueous solution onto viscose-based activated carbon fiber felts: kinetics and equilibrium studies. *Adsorption Science & Technology*.

[28] Yang J., Qiu K. (2010). Preparation of activated carbons from walnut shells via vacuum chemical activation and their application for methylene blue removal. *Chemical Engineering Journal*.

[29] Elmouwahidi A., Zapata-Benabithe Z., Carrasco-Marín F., Moreno-Castilla C. (2012). Activated carbons from KOH-activation of argan (Argania spinosa) seed shells as supercapacitor electrodes. *Bioresource Technology*.

[30] Gumus R. H., Okpeku I. (2014). Production of activated carbon and characterization from snail shell waste (Helix pomatia). *Advances in Chemical Engineering and Science*.

[31] Moubarik A., Grimi N. (2015). Valorization of olive stone and sugar cane bagasse by-products as biosorbents for the removal of cadmium from aqueous solution. *Food Research International*.

[32] Guechi E.-K., Hamdaoui O. (2016). Sorption of malachite green from aqueous solution by potato peel: kinetics and equilibrium modeling using non-linear analysis method. *Arabian Journal of Chemistry*.

[33] Azmi N. B., Bashir M. J. K., Sethupathi S., Ng C. A. (2016). Anaerobic stabilized landfill leachate treatment using chemically activated sugarcane bagasse activated carbon: kinetic and equilibrium study. *Desalination and Water Treatment*.

[34] Heidarinejad Z., Rahmanian O., Fazlzadeh M., Heidari M. (2018). Enhancement of methylene blue adsorption onto activated carbon prepared from Date Press Cake by low frequency ultrasound. *Journal of Molecular Liquids*.

[35] Cundari L., Sari K. F., Anggraini L. (2018). Characteristic of betel nuts activated carbon and its application to Jumputan wastewater treatment. *Materials Science and Engineering*.

[36] El Maguana Y., Elhadiri N., Bouchdoug M., Benchanaa M., Jaouad A. (2019). Activated carbon from prickly pear seed cake: optimization of preparation conditions using experimental design and its application in dye removal. *International Journal of Chemical Engineering*.

[37] Benabid A. (2000). *Flore et écosystème du Maroc: évaluation et préservation de la biodiversité*.

[38] Ionesco T., Sauvage C. (1969). Fichier des espèces climax. *Al Awamia*.

[39] Damiyine B., Guenbour A., Boussen R. (2017). Adsorption of rhodamine B dye onto expanded perlite from aqueous solution: kinetics, equilibrium and thermodynamics. *Journal of Materials and Environmental Science*.

[40] Fayoud N., Younssi S. A., Tahiri S., Albizane A. (2015). Kinetic and thermodynamic study of the adsorption of methylene blue on wood ashes. *Journal of Materials and Environmental Science*.

[41] Das B., Mondal N. K., Bhaumik R., Roy P., Pal K. C., Das C. R. (2013). Removal of copper from aqueous solution using alluvial soil of Indian origin: equilibrium, kinetic and thermodynamic study. *Journal of Materials and Environmental Science*.

[42] Raoul T. T. D., Gabche A. S., Mbadcam K. J., Ndifor-Angwafor N. G., Nsami N. J. (2014). Kinetics and equilibrium studies of adsorption of phenol in aqueous solution onto activated carbon prepared from rice and coffee husks. *International Journal of Engneering and Technological Research*.

[43] Ojemaye M. O., Okoh O. O., Okoh A. I. (2017). Adsorption of Cu^2+^ from aqueous solution by a novel material; azomethine functionalized magnetic nanoparticles. *Separation and Purification Technology*.

[44] Langmuir I. (1918). The adsorption of gases on plane surfaces of glass, mica and platinum. *Journal of the American Chemical Society*.

[45] Ali R. M., Hamad H. A., Hussein M. M., Malash G. F. (2016). Potential of using green adsorbent of heavy metal removal from aqueous solutions: adsorption kinetics, isotherm, thermodynamic, mechanism and economic analysis. *Ecological Engineering*.

[46] Choudhary M., Kumar R., Neogi S. (2020). Activated biochar derived from Opuntia ficus-indica for the efficient adsorption of malachite green dye, Cu^+2^ and Ni^+2^ from water. *Journal of Hazardous Materials*.

[47] Ayawei N., Ebelegi A. N., Wankasi D. (2017). Modelling and interpretation of adsorption isotherms. *Journal of Chemistry*.

[48] Freundlich H. M. F. (1906). Over the adsorption in solution. *Journal of Physical Chemistry*.

[49] Nourmoradi H., Avazpour M., Ghasemian N. (2016). Surfactant modified montmorillonite as a low cost adsorbent for 4-chlorophenol: equilibrium, kinetic and thermodynamic study. *Journal of the Taiwan Institute of Chemical Engineers*.

[50] Kernels F. M. S. (2018). Adsorption du méthylorange sur un biosorbant à base de noyaux de mangue. *Larhyss Journal*.

[51] Mekhalef Benhafsa F., Kacha S., Leboukh A., Belaid K. D. (2018). Étude comparative de l’adsorption du colorant Victoria Bleu Basique à partir de solutions aqueuses sur du carton usagé et de la sciure de bois. *Revue des Sciences de l’Eau*.

[52] Okman I., Karagöz S., Tay T., Erdem M. (2014). Activated carbons from grape seeds by chemical activation with potassium carbonate and potassium hydroxide. *Applied Surface Science*.

[53] Abdel-Ghani N. T., El-Chaghaby G. A., ElGammal M. H., Rawash E.-S. A. (2016). Optimizing the preparation conditions of activated carbons from olive cake using KOH activation. *New Carbon Materials*.

[54] Sahu J. N., Acharya J., Meikap B. C. (2010). Optimization of production conditions for activated carbons from Tamarind wood by zinc chloride using response surface methodology. *Bioresource Technology*.

[55] Shams Khorramabadi G., Darvishi Cheshmeh Soltani R., Rezaee A., Khataee A. R., Jonidi Jafari A. (2012). Utilisation of immobilised activated sludge for the biosorption of chromium (VI). *Canadian Journal of Chemical Engineering*.

[56] Zhong Z.-Y., Yang Q., Li X.-M., Luo K., Liu Y., Zeng G.-M. (2012). Preparation of peanut hull-based activated carbon by microwave-induced phosphoric acid activation and its application in Remazol Brilliant Blue R adsorption. *Industrial Crops and Products*.

[57] Liou T.-H. (2010). Development of mesoporous structure and high adsorption capacity of biomass-based activated carbon by phosphoric acid and zinc chloride activation. *Chemical Engineering Journal*.

[58] Saka C. (2012). BET, TG-DTG, FT-IR, SEM, iodine number analysis and preparation of activated carbon from acorn shell by chemical activation with ZnCl_2_. *Journal of Analytical and Applied Pyrolysis*.

[59] Chen C.-X., Yang S.-S., Ding J. (2021). Non-covalent self-assembly synthesis of AQ2S@rGO nanocomposite for the degradation of sulfadiazine under solar irradiation: the indispensable effect of chloride. *Applied Catalysis B: Environmental*.

[60] Angin D. (2014). Production and characterization of activated carbon from sour cherry stones by zinc chloride. *Fuel*.

[61] Liu W., Li J., Zheng J. (2020). Different pathways for Cr(III) oxidation: implications for Cr(VI) reoccurrence in reduced chromite ore processing residue. *Environmental Science & Technology*.

[62] Liu J., Zhang Q., Tian X. (2021). Highly efficient photocatalytic degradation of oil pollutants by oxygen deficient SnO2 quantum dots for water remediation. *Chemical Engineering Journal*.

[63] Li G., Huang S., Zhu N., Yuan H., Ge D., Wei Y. (2021). Defect-rich heterojunction photocatalyst originated from the removal of chloride ions and its degradation mechanism of norfloxacin. *Chemical Engineering Journal*.

[64] Miraboutalebi S. M., Nikouzad S. K., Peydayesh M., Allahgholi N., Vafajoo L., McKay G. (2017). Methylene blue adsorption via maize silk powder: kinetic, equilibrium, thermodynamic studies and residual error analysis. *Process Safety and Environmental Protection*.

[65] Reza R. A., Ahmaruzzaman M. (2015). A novel synthesis of Fe2O3@activated carbon composite and its exploitation for the elimination of carcinogenic textile dye from an aqueous phase. *RSC Advances*.

[66] Tan I. A. W., Hameed B. H. (2010). Adsorption isotherms, kinetics, thermodynamics and desorption studies of basic dye on activated carbon derived from oil palm empty fruit bunch. *Journal of Applied Sciences*.

[67] Yang X., Li F., Xia M., Luo F., Jiang Y. (2018). Investigation on the micro-structure and adsorption capacity of cellulosic biomass carbon based montmorillonite composite. *Microporous and Mesoporous Materials*.

[68] Ajmal A., Piergiovanni P. R. (2018). Effect of mordanting on the adsorption thermodynamics and kinetics of cochineal for wool. *Industrial & Engineering Chemistry Research*.

[69] Touzani I., Fikri-Benbrahim K., Ahlafi H., Ihssane B., Boudouch O. (2021). Characterization of activated carbon prepared from then nucleus of Ziziphus lotus (Nbeg): isothermal study and methylene blue’s adsorption kinetics. https://www.researchsquare.com/article/rs-619904/v1.

