# Supplementary material for: Characterization of Ziziphus lotus' Activated Carbon and Evaluation of Its Adsorption Potential
Source: J Environ Public Health. 2022 Apr 25;2022:8502211. doi: 10.1155/2022/8502211 (PMC9061054; doi:10.1155/2022/8502211)
Supplement: Supplementary Materials — We point out that it concerns an international patent including only a part of the results presented in our article and concerning the characterization of activated carbon only by electronic microscopy, diffraction of RX, and infrared red spectrum, with the reduction of methylene blue. The paper also includes the specific surface by the BET method and the effect of temperature, besides kinetic and thermodynamic studies of methylene blue adsorption and the Langmuir and Freundlich isotherms to determine the adsorption nature. The OMPIC file also concerns a national patent (presented in Morocco), including the same parameters presented for the WIPO one, and can be searched in the link of OMPIC (https://patent.ompic.ma/search) by giving the number of the patent (MA 49232). [file 8502211.f1.zip › 8502211.f1/WO2021182941A2.pdf]

(12) DEMANDE INTERNATIONALE PUBLIÉE EN VERTU DU TRAITÉ DE COOPÉRATION EN MATIÈRE DE BREVETS (PCT)

(19) **Organisation Mondiale de la Propriété Intellectuelle**
Bureau international

(43) **Date de la publication internationale**
16 septembre 2021 (16.09.2021)

WIPO | PCT

(10) **Numéro de publication internationale**
WO 2021/182941 A2

(51) **Classification internationale des brevets :** Non classée

(21) **Numéro de la demande internationale :** PCT/MA2021/050001

(22) **Date de dépôt international :** 12 mars 2021 (12.03.2021)

(25) **Langue de dépôt :** français

(26) **Langue de publication :** français

(30) **Données relatives à la priorité :**
49232 13 mars 2020 (13.03.2020) MA

(71) **Déposant : UNIVERSITÉ SIDI MOHAMED BEN ABDELLAH** [MA/MA] ; Route Imouzzer BP 2626 FES 30000, Fès, 30000 (MA).

(72) **Inventeurs : TOUZANI, Ibrahim** ; Universite Sidi Mohamed Ben Abdellah BP 2626 Fes (MA), Faculte Des Sciences Et Techniques De Fes, B.P. 2202 Maroc, Fès, 30000 (MA). **FIKRI BENBRAHIM, Kawtar** ; Universite Sidi Mohamed Ben Abdellah BP 2626 Fes (MA), Faculte Des Sciences Et Techniques De Fes, B.P. 2202 Maroc, Fès, 30000 (MA). **BOUDOUCH, Otmane** ; Universite Sultan Moulay Slimane BP 591 Beni Mellal (MA), Faculte Des Sciences Et Techniques, B.P : 523, Beni - Mellal (MA). **ELKACMI, Reda** ; Universite Sultan Moulay Slimane BP 591, Faculte Des Sciences Et Techniques, Beni Mellal (MA).

(74) **Mandataire : IBNSOUDA, Saad** ; Route Imouzzer Fès BP 2626, Fès, 30000 (MA).

(81) **États désignés** *(sauf indication contraire, pour tout titre de protection nationale disponible)* : AE, AG, AL, AM, AO, AT, AU, AZ, BA, BB, BG, BH, BN, BR, BW, BY, BZ, CA, CH, CL, CN, CO, CR, CU, CZ, DE, DJ, DK, DM, DO, DZ, EC, EE, EG, ES, FI, GB, GD, GE, GH, GM, GT, HN, HR, HU, ID, IL, IN, IR, IS, IT, JO, JP, KE, KG, KH, KN, KP, KR, KW, KZ, LA, LC, LK, LR, LS, LU, LY, MA, MD, ME, MG, MK, MN, MW, MX, MY, MZ, NA, NG, NI, NO, NZ, OM, PA, PE, PG, PH, PL, PT, QA, RO, RS, RU, RW, SA, SC, SD, SE, SG, SK, SL, ST, SV, SY, TH, TJ, TM, TN, TR, TT, TZ, UA, UG, US, UZ, VC, VN, WS, ZA, ZM, ZW.

(84) **États désignés** *(sauf indication contraire, pour tout titre de protection régionale disponible)* : ARIPO (BW, GH, GM, KE, LR, LS, MW, MZ, NA, RW, SD, SL, ST, SZ, TZ, UG, ZM, ZW), eurasien (AM, AZ, BY, KG, KZ, RU, TJ, TM), européen (AL, AT, BE, BG, CH, CY, CZ, DE, DK, EE, ES, FI, FR, GB, GR, HR, HU, IE, IS, IT, LT, LU, LV, MC, MK, MT, NL, NO, PL, PT, RO, RS, SE, SI, SK, SM, TR), OAPI (BF, BJ, CF, CG, CI, CM, GA, GN, GQ, GW, KM, ML, MR, NE, SN, TD, TG).

Publiée:
— *sans rapport de recherche internationale, sera republiée dès réception de ce rapport (règle 48.2(g))*
— *en noir et blanc ; la demande internationale telle que déposée était en couleur ou en échelle de gris et est disponible sur PATENTSCOPE pour téléchargement.*

(54) **Title:** ACTIVATED CARBON PREPARED USING THE KERNEL OF THE JUJUBE *ZIZIPHUS LOTUS* (SIDR) AND METHOD FOR MANUFACTURING SAME

(54) **Titre :** CHARBON ACTIF PRÉPARÉ A BASE DU NOYAU DU NBEG *ZIZIPHUS LOTUS* (SIDR) ET SON PROCÉDÉ DE FABRICATION

(57) **Abstract:** The present invention relates to an activated carbon prepared using a new biomaterial and to the method for the manufacture thereof based on the chemical/thermal activation of the kernel of the JUJUBE ZIZIPHUS LOTUS (sidr) and the use thereof as a new adsorption support for wastewater treatment, biogas purification, abatement of metallic trace elements, reduction of the organic and microbial load.

(57) **Abrégé :** La présente invention concerne un charbon actif prépare a base d'un nouveau biomatériau et son précédé de fabrication base sur l'activation chimique/thermique du noyau du NBEG ZIZIPHUS LOTUS (Sidr) et son application comme un nouveau support d'adsorption pour le traitement des eaux usées, la purification du biogaz, abattement des éléments de trace métalliques, la réduction de la charge organique et microbienne.

WO 2021/182941 A2

Titre : charbon actif préparé a base du noyau du Nbeg *Ziziphus Lotus* (sidr) et son procédé de fabrication

Description

Domaine de l'invention

La présente invention concerne un nouveau matériau qui est le charbon actif, qui peut être appliqué pour l'épuration des eaux résiduaires urbaines, industrielles et des lixiviats, pour lequel nous avons évalué les potentialités d'adsorption sur le bleu de méthylène (CI 52015).

Etat de la technique antérieure

Au début du 20ème siècle, les méthodes de fabrication s'améliorent et certains procédés sont mis au point pour augmenter les performances de ces matériaux. Cette révolution ayant comme point de départ des travaux d'Ostreijko (R. Von Ostreijko, Patents 1900-1901), qui a découvert les deux méthodes de base de la production du charbon actif à savoir l'activation chimique et l'activation physique.

M. lopez et al., (1996) ont décrit un procédé qui consiste à imprégner uniformément un précurseur de charbon (bois, coque d'amande ou de noix de coco, …) par trempage dans une solution aqueuse à 80°C contenant un agent activant (H_3PO_4, $ZnCl_2$). Le précurseur imprégné est ensuite séché et / ou directement activé à une température de l'ordre de 500°C pendant une heure. L'agent activant résiduel est ensuite éliminé par rinçage à l'eau. Le temps nécessaire pour la phase d'imprégnation est cependant très élevé, ce qui limite l'intérêt industriel et économique du procédé.

Le brevet MA 33642 B1, décrit un procédé de préparation du charbon actif à partir de la farine des coquilles d'œuf, destiné comme un nouveau support catalytique en synthèse organique hétérogène solide-liquide. Les coquilles d'œufs sont lavées plusieurs fois avec de l'eau du robinet et laissées à l'air libre pendant plusieurs jours ; puis séchées à l'étuve à 80°C. Les coquilles séchées sont broyées en petites particules de taille micro millimétrique jusqu'à 250 µm. Le matériau obtenu est calciné dans une gamme de température de 400 à 800°C pendant une durée variable qui peut atteindre jusqu'à 2h. Ce matériau calciné est broyé et relavé encore une fois avec de l'eau puis séché à l'étuve à une température comprise entre 60 et 80°C. Par la

suite le matériau séché est calciné dans un four à une vitesse de chauffage variable de 1 à 4°C/min à 400°C de 2 à 5h.

Le brevet MA 33786 B1, expose un procédé de préparation d'un charbon actif à base de noix d'argan en utilisant l'acide phosphorique (H_3PO_4).

Le brevet FR3009789A1, décrit une méthode permettant de contrôler l'abattement des micropolluants organiques dans les eaux usées lors du traitement par adsorption sur charbon actif. Cette méthode comprend la mesure de l'absorbance, à une longueur d'onde donnée A, des eaux usées avant et après traitement par adsorption sur charbon actif et la détermination du pourcentage d'abattement des micropolluants organiques à partir du pourcentage de diminution de l'absorbance calculé.

Le brevet CN108766773 A, présente un procédé d'élaboration d'un charbon actif à base d'algues comprenant le broyage des algues dans un moulin colloïdal, le séchage et le tamisage, la carbonisation, le lavage et l'activation des algues séchées pour obtenir un produit activé, ainsi le lavage et le séchage du produit activé.

Le brevet CN103406094 A révèle une méthode de fabrication de charbon actif à base des boues redondantes dans une station d'épuration urbaine. Le procédé comprend les étapes consistant à immerger la boue séchée redondante dans du chlorure ferrique pour une activation pendant 24 heures, dégoulinant l'eau d'ammoniac, le séchage à une température de 105°C, carbonisation et activation du mélange dans un four à silencieux, nettoyage du mélange avec de l'eau distillée, les produits de broyage et de criblage et l'exécution de la séparation magnétique pour obtenir le carbone actif à base de boues magnétiques.

Le brevet CN102745689 A, rapporte une méthode de préparation du carbone activé par un champignon microbien de pourriture blanche ou une activation catalysée enzymatique du tabac basé sur la méthode existante pour la préparation du carbone activé. Selon différents procédés de préparation, les déchets solides de tabac sont utilisés comme matière première, et des enzymes d'ingénierie (cellulase et ligninase) ou des microorganismes (champignons de la pourriture blanche) sont sélectionnés comme activateurs pour catalyser l'activation de la lignocellulose et la catalyse enzymatique des matières premières. L'hydrolyse favorise la formation de structures lâches dans la matière première carbonisée.

Exposé de l'invention

En 1974, Le charbon actif a été le premier adsorbant exploité industriellement pour la décoloration du sirop de sucre en Angleterre. Par la suite plusieurs recherches ont été menées pour améliorer les propriétés d'adsorption de ce type de matériau.

Selon le conseil Européen des Fédérations de l'Industrie Chimique, les charbons actifs, sont des produits carbonés dotés d'une structure poreuse présentant une très grande surface de contact interne, ces matériaux issus d'un procédé industriel peuvent adsorber une large variété de substances.

Les substrats les plus couramment utilisés à l'échelle commerciale pour la fabrication du charbon sont : le bois, l'anthracite, le charbon de bitume, le lignite, les coquilles de noix de coco, les grignions d'olive ainsi que les coquilles d'amande. Aujourd'hui, beaucoup d'efforts sont consacré à l'exploitation des déchets industriels et les résidus agricoles en tant que matières premières de production de charbon. Parmi ses produits on trouve les tiges de maïs, rouleaux de riz, bagasse, coquilles (pistache, amande et noix de pécan), Noyaux de palmier, pulpe de fruits et grains de café (Crini et al., 2019).

La principale caractéristique d'un charbon actif est sa structure poreuse, à savoir la surface spécifique accessible pour telle ou telle molécule sonde, la distribution de la taille des pores et leur forme géométrique moyenne. Le charbon actif en poudre (CAP) prend la forme de grains de taille comprise entre 50 et 100 µm.

Les propriétés du charbon actif dépendent principalement du type d'activateur (Sawant et al., 2017 ; Uysal et al., 2014). Dans ce sens la sélection d'agents d'activation reste un enjeu clé pour de nombreux chercheurs. Dans la littérature, il existe de nombreux articles scientifiques qui rapportent l'activation avec de l'hydroxyde de potassium, l'acide phosphorique, chlorure de zinc, carbonate de potassium, l'hydroxyde de sodium et de nouveaux agents d'activation. L'activation avec l'acide phosphorique est couramment utilisée pour les matières ligno-cellulosiques. Le chlorure de zinc génère plus de surface que l'acide phosphorique mais il est moins utilisé en raison de préoccupations environnementales. Le carbonate de potassium, par rapport à l'hydroxyde de potassium, produit des rendements plus élevés et une surface aussi plus élevée pour l'adsorption de grosses molécules polluantes telles que les colorants. L'activation avec l'hydroxyde de potassium en termes de surface et d'efficacité donne de meilleurs résultats que l'hydroxyde de sodium pour diverses applications (Zoha Heidarinejad et *al*., 2020).

La présente invention concerne la préparation d'un charbon actif à base d'un nouveau biomatériau, par l'activation chimique du noyau du Nbeg *Ziziphus lotus*(NZL). Ce charbon actif possède une porosité très développée qui s'étend de 10 à 45µm. De plus, sa structure superficielle possède des groupements acides et des fonctions carboxyliques qui augmentent son pouvoir d'adsorption et sa capacité d'abattement sur un grand nombre d'effluents.

Un autre volet de l'invention concerne l'utilisation de ce charbon actif comme un nouveau support pour la dépollution des eaux usées urbaines et industrielles chargées en polluants organiques et métalliques. Un exemple d'application est l'étude d'adsorption du bleu de méthylène CI 52015 qui a donné un taux d'abattement qui atteint 97% après une heure de contact.

Brève description des figures

La figure 1 présente une image de Microscope Electronique à Balayage du charbon actif préparé à partir du noyau du NZL.

La figure 2 présente le diagramme de diffraction des rayons X du charbon actif préparé à partir du noyau du NZL.

La figure 3 présente l'analyse par spectroscopie infrarouge à transformée de Fourier du charbon actif préparé à partir du noyau du NZL.

La figure 4 présente la structure chimique du bleu de méthylène CI 52015

La figure 5 présente le pourcentage d'abattement du bleu de méthylène en fonction du temps par adsorption sur le charbon actif préparé à partir du noyau du NZL.

Exposé détaillé de l'invention

L'invention concerne la préparation d'un charbon actif à base d'un nouveau biomatériau, par l'activation chimique du noyau du Nbeg *Ziziphus lotus* (NZL).

La figure (1) qui représente une image du charbon actif, produit par activation du noyau de NZL, observée par microscopie électronique à balayage (MEB), montre une morphologie très poreuse du charbon actif avec des pores de différentes tailles allant de 10 à 45µm et de différentes formes, qui prouvent que le charbon actif à une grande surface spécifique. De même la diffraction des rayons X (DRX) du charbon actif présenté dans la figure (2), montre une structure amorphe avec des pics indiquant la présence du carbone et de la cellulose. Ainsi Le

spectre infrarouge du charbon actif présenté dans la figure (3), indique que le charbon actif présente différents groupes fonctionnels tels que des groupements hydroxyle, carboxyle et carbonyle, qui peuvent être des sites potentiels d'adsorption.

La préparation de notre charbon actif suit plusieurs étapes. Les noyaux NZL sont lavés plusieurs fois puis séchés à l'ombre et à l'abri de la poussière, ensuite ils sont broyés en fines particules. L'activation chimique est effectuée par un acide notamment l'acide sulfurique (H_2SO_4, 98%). Ensuite, la carbonisation de la pâte obtenue est conduite à une température de 500°C. Le charbon actif ainsi obtenu est broyé en petites particules de tailles inférieures à 100µm et nommé CAPNZL.

Le taux d'humidité est mesuré en pesant une quantité du CAPNZL avant et après séchage à 110°C. Le taux d'humidité obtenu est de 2,9%.

Le taux de cendre (TC) est mesuré en pesant la masse finale suite à l'incinération à 600°C d'un gramme du CAPNZL suivie d'un refroidissement. La teneur en cendres est de 4,2%.

Exemple d'application du CAPNZL : Adsorption du colorant CI 52015

Les colorants sont utilisés dans de nombreux secteurs industriels tels que les teintures du textile, du papier, du cuir et dans les industries alimentaires et cosmétiques. Les colorants ont la réputation d'être des substances toxiques et persistantes dans l'environnement.

Le bleu de méthylène est le colorant le plus couramment utilisé dans la teinture du coton, du bois et de la soie. Il peut provoquer des brûlures oculaires responsables de blessures permanentes aux yeux de l'homme et des animaux. Son inhalation peut provoquer des difficultés respiratoires et son ingestion par la bouche produit une sensation de brûlure. Le traitement des rejets industriels contenant ce type de colorant s'avère d'un grand intérêt.

Le bleu de méthylène (figure 4) est un colorant cationique dont le Colour Index « CI » est 52015, la formule est $C_{16}H_{18}N_3SC_1$ et la masse molaire est de 319,85mol/g.

Dans un réacteur fermé, un volume de bleu de méthylène CI52015 à différentes concentrations est mis en contact avec une masse de charbon actif CAPZL, puis la solution est agitée à température ambiante. Après chaque 10 min de contact, la suspension est centrifugée, les concentrations du CI52015 dans les surnageants sont mesurées à l'aide de la spectrophotométrie UV-Visible.

Les résultats d'adsorption (Figure5) montrent que le processus d'adsorption est très rapide, en effet, plus de 80 % de la quantité utilisée du colorant est adsorbée pendant les vingt premières

minutes. Ce qui signifie que notre charbon actif préparé à partir du noyau du NZL est un bon adsorbant et pourra être utilisé dans les procédés de traitement des eaux usées, lixiviats et pour la purification du biogaz etc...

Application industrielle

La présente invention concerne un procédé de fabrication du charbon actif à base du noyau du Nbeg *Ziziphus lotus*. Le charbon actif produit à partir dudit procédé peut être utilisé pour le traitement des eaux usées, des lixiviats et la purification des biogaz.

Revendications

1. Procédé de fabrication du charbon actif, caractérisé en ce qu'il utilise le noyau du Nbeg *Ziziphus lotus* (sidr) comme matière première.
2. Procédé de fabrication du charbon actif, selon la revendication 1, caractérisé en ce qu'il comporte les étapes suivantes :
   - Etape 1 : lavage et séchage du noyau du Nbeg *Ziziphus lotus ;*
   - Etape 2 : broyage en fines particules du noyau du Nbeg *Ziziphus lotus ;*
   - Etape 3 : activation chimique du noyau du Nbeg *Ziziphus lotus* par un acide ;
   - Etape 4 : carbonisation de la pâte obtenue après l'activation chimique ;
   - Etape 5 : broyage et tamisage du charbon actif obtenu après la carbonisation.
3. Procédé de fabrication du charbon actif, selon les revendications 1 et 2, caractérisé en ce que l'étape de l'activation est faite avec l'acide sulfurique ;
4. Procédé de fabrication du charbon actif, selon les revendications 1 et 2, caractérisé en ce que l'étape de carbonisation est faite à une température de 500°C pendant 2 h ;
5. Procédé de fabrication du charbon actif, selon les revendications 1 et 2, caractérisé en ce que l'étape de tamisage est faite avec un tamis de taille inférieur à 100µm.
6. Charbon actif produit à partir du procédé de fabrication selon les revendications de 1 à 5, caractérisé en ce qu'il présente une porosité élevée avec une taille de pore allant de 10 à 45µm.
7. Charbon actif selon la revendication 6, caractérisé en ce qu'il peut être utilisé pour traiter les effluents à forte charge organique.

Dessins

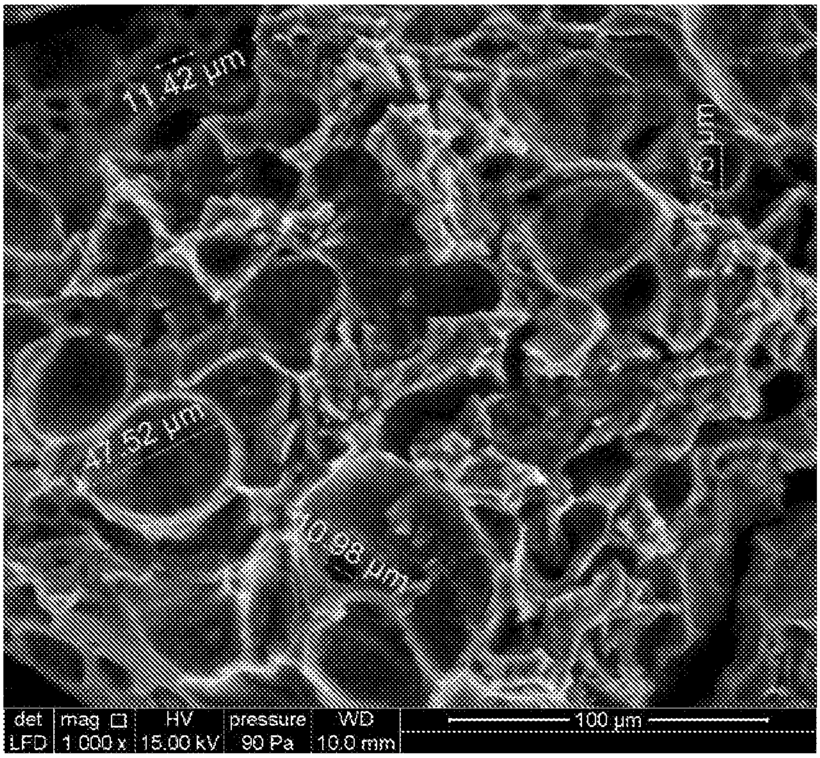

Fig1 : Image du CAPNZL observé par microscopie électronique à balayage (MEB)

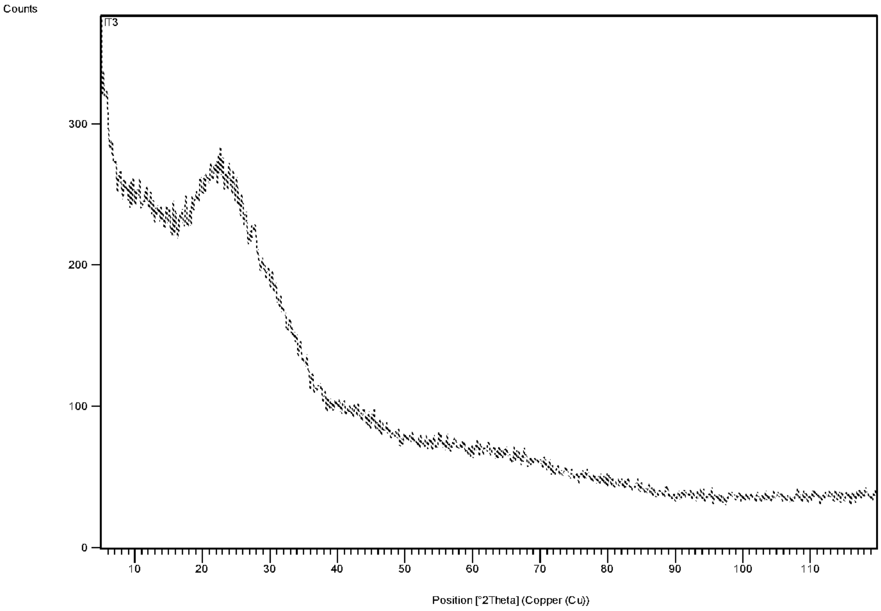

Fig 2 : Diffraction des rayons X du CAPNZL

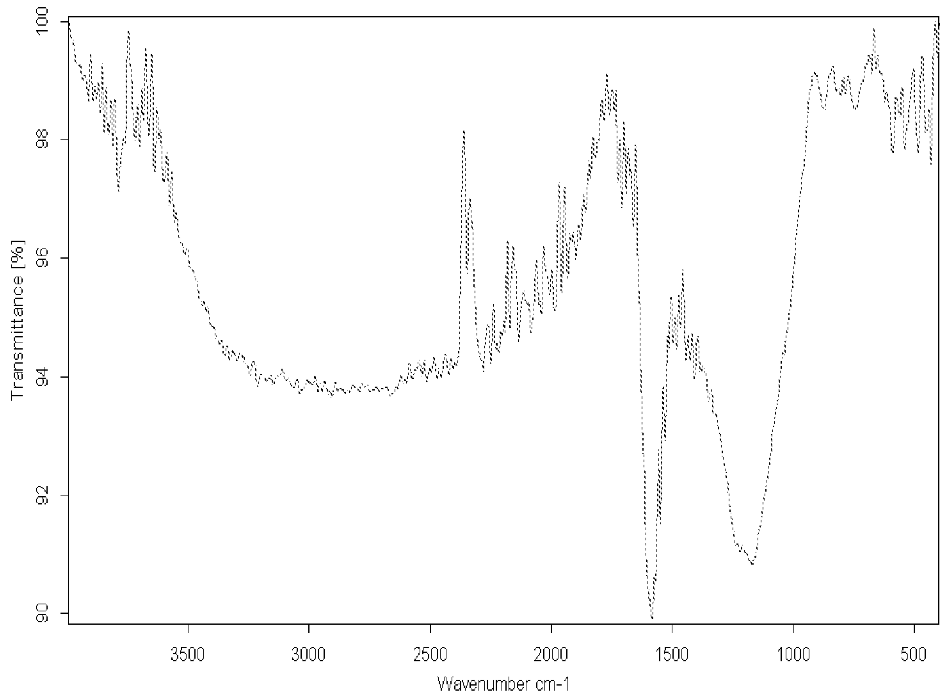

__Fig 3 : Spectre IR du CAPNZL__

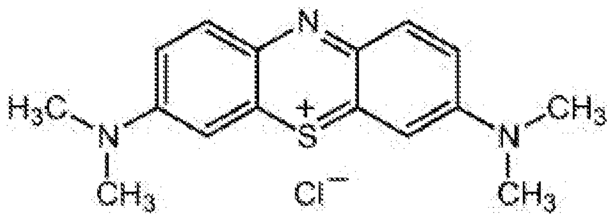

__Fig4 : Structure moléculaire du CI 52015__

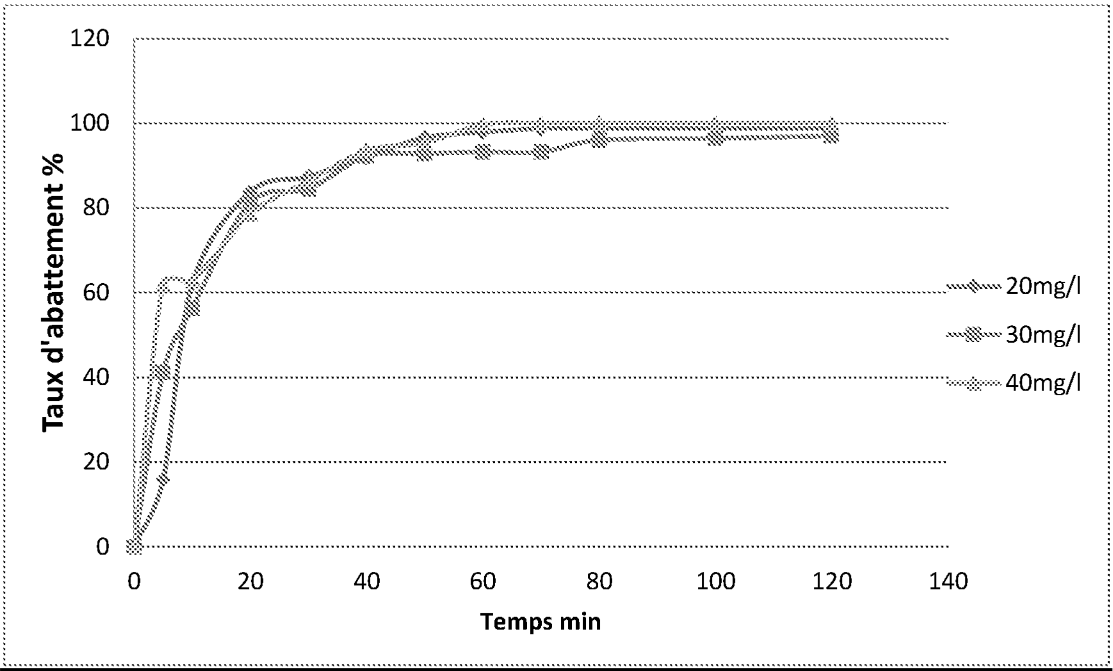

Fig 5 : Taux d'abattement du CI 52015 par CAPNZL
